# Exploring the connection between caffeine intake and constipation: a cross-sectional study using national health and nutrition examination survey data

**DOI:** 10.1186/s12889-023-17502-w

**Published:** 2024-01-02

**Authors:** Yulong Kang, Jin Yan

**Affiliations:** Department of Proctology, Northern Jiangsu People’s Hospital, No.98 Nantong Western Road, Guangling District, Yangzhou, 225001 P.R. China

**Keywords:** Caffeine consumption, Constipation management, Middle-age people, Decaffeinated coffee

## Abstract

**Background:**

Caffeine has been reported to increase gastrointestinal motility and change intestinal microbiota. Constipation may be caused by colonic motor dysfunction and colonic microbiomeis disturbances. In this study, we aimed to explore the association between caffeine intake and constipation.

**Methods:**

This was a cross-sectional study based on the National Health and Nutrition Examination Survey (NHANES). Caffeine intake was assessed using 24-h dietary recall method, and constipation was defined based on stool consistency or stool frequency. Logistic regression analysis was used to assess the association between caffeine intake and constipation, and results were expressed as odds ratio (OR) with 95% confidence intervals (95%CI). Subgroup analysis was performed based on age.

**Results:**

A total of 13,816 participants were finally included for analysis. After adjusting potential confounders, high intake of caffeine was found to be associated with the low odds of constipation (Q3: OR = 0.60, 95%CI: 0.49–0.74; Q4: OR = 0.77, 95%CI: 0.59–0.99; Q5: OR = 0.72, 95%CI: 0.56–0.92). The similar association was found in young people and middle-age people (*P* < 0.05).

**Conclusion:**

High caffeine intake was associated with the low odds of constipation. Our finding indicated that individuals should develop consciousness and habit of consuming caffeinated foods and drinks to prevent and relief the constipation.

**Supplementary Information:**

The online version contains supplementary material available at 10.1186/s12889-023-17502-w.

## Background

Constipation is characterized by fewer than three bowel movements per week, with hard, dry, or lumpy stools [1]. The prevalence of constipation ranges from 7 to 10% in the adults and varies by age [2, 3]. Constipation leads to anxiety, depression, and cognitive impairment, which seriously affect people’s quality of life [4, 5]. In addition, constipation increases the medical burden that the annual costs of constipation treatment are estimated to exceed $230 million in the United States [6]. Generally, dietary adjustment is considered for constipation prior to medical intervention for the purpose of minimizing side effects of some drugs [7]; therefore, some dietary factors have been found to be associated with constipation [8, 9].

Coffee is a worldwide popular beverage, and caffeine is a major water-soluble component of coffee [10]. Coffee has been reported to play a beneficial role in human health, which reduces the risk of cardiovascular diseases, neurodegenerative diseases, and metabolic syndrome [10, 11]. Some studies have reported the regulatory effect of coffee on the colonic motor function and gut microbiota [12–14]. Gkegkes et al. have found that consuming coffee in the postoperative period significantly decreased the time to first bowel movement, the time to first flatus, and the time to tolerance of solid diet [12]. Hussain et al. have reported that coffee consumption was associated with intestinal microbial diversity, and there was a dose-response association between coffee consumption and relative abundance of Veillonella [15]. Coffee consumption appears to be associated with changes of some gut microbiota in which caffeine may play a role [13]. An animal model has displayed that caffeine might regulate the gut microbial community to repair the disrupted short-chain fatty acids profile [16]. Colonic motor dysfunctions and colonic microbioma disturbances may be the possible reasons for constipation [6]. However, the association between caffeine intake and constipation has not been very well investigated.

### Objective

In this study, we aimed to explore the association between caffeine intake and constipation. This study may be contributed to expand the existing knowledge of physiological effect of caffeine on the gastrointestinal tract to help clinicians and people in the management of constipation.

## Methods

### Study design and data source

This was a cross-sectional study, and data were extracted from the National Health and Nutrition Examination Survey (NHANES) database. Our study was a secondary data analysis, and the National Center for Health Statistics (NCHS) collected the data. NHANES was a project to assess health and nutritional status of adults and children in the United States. Fifteen counties across the country and a nationally representative sample of about 5,000 persons were examined every year. This survey combined interviews and physical examinations. Ethical approval from the Institutional Review Board of Northern Jiangsu People’s Hospital and informed consent of the subjects were not needed because NHANES was a publicly available database. All methods were carried out in accordance with relevant guidelines and regulations.

### Study population

Participants were extracted from 2005 to 2010 NHANES database because these three 2-year cycles (2005–2006, 2007–2008, 2009–2010) recorded the data on constipation. Participants met all the following criteria were included: (1) age ≥ 20 years; (2) with data on stool consistency and frequency; and (3) with data on caffeine intake. Participants met one of the following criteria were excluded: (1) pregnant women; (2) self-reporting the history of colon cancer, celiac disease or inflammatory bowel disease.

### Caffeine intake

Caffeine intake was obtained from the 24-hour dietary recall interview, and caffeine included the intake of the sum of food, beverages, and dietary supplements. Supplementary Fig. 1 demonstrates the source and proportion of caffeine intake. Participants were requested to recall all the food and beverages consumed in the past 24 h (midnight to midnight). Two 24-hour dietary recall interviews were conducted. The first dietary recall interview was conducted in-person in the Mobile Examination Center (MEC), and the second interview was conducted by telephone 3 to 10 days later [17]. Dietary data were collected using a computer-assisted food coding, and day-to-day variations were accounted using the U.S Department of Agriculture (USDA) Automated Multiple-Pass Method [17]. USDA’s Food and Nutrient Database for Dietary Studies (FNDDS) provided the nutritional values of all food and beverages, and was regularly updated each cycle [17]. In this study, we used the reported nutritional value in the first dietary recall interview. For dietary supplements, participants were asked whether they used any dietary supplements in the past 30 days during an in-house interview; someone who answered “Yes” was further asked about the product name, frequency, duration, and serving form [18]. For each nutrient, the daily dose was calculated by combining the frequency with the product information on ingredient, amount of ingredient per serving, and ingredient unit [18]. Nutrient intake from each product was summed to assess the total daily dose of each supplemental nutrient for an individual [18]. According to quintiles, caffeine intake was divided into five groups: Q1 (≤ 10 mg), Q2 (10–86 mg), Q3 (86–171 mg), Q4 (171–303 mg), and Q5 (> 303 mg).

The intake of coffee, caffeinated coffee, and decaffeinated coffee was also ascertained from the 24-hour dietary recall. Coffee intake, caffeinated coffee intake, and decaffeinated coffee intake were divided into five groups, but quintiles could not be used to divide them because 46.73% of the participants reported no coffee intake, 53.86% of the participants reported no caffeinated coffee intake, and 91.44% of the participants reported no decaffeinated coffee intake. Beyond those who reporting no intake of coffee/caffeinated coffee/decaffeinated coffee, participants were divided into quartiles, resulting in five categories. Coffee intake was divided into no coffee intake (0 g), 0-311 g, 311–502 g, 502–754 g, and > 754 g. Caffeinated coffee intake was divided into no caffeinated coffee intake (0 g), 0-310 g, 310–502 g, 502–754 g, and > 754 g. Decaffeinated coffee intake was divided into no decaffeinated coffee intake (0 g), 0-250 g, 250–357 g, 357–700 g, and > 754 g.

### Constipation definition

Constipation was defined based on stool consistency or stool frequency according to NHANES database [19, 20]. Stool consistency was estimated using the Bristol stool form scale, which contained a variety of colorful cards and detailed descriptions of seven stool types. When be asked “Please look at this card and tell me the number that corresponds with your usual or most common stool type”, participants who answered Type 1 (separate hard lumps, like nuts) or Type 2 (sausage-like, but lumpy) were considered to suffer from constipation [21]. Stool frequency was estimated with the following question: “How often have bowel movements?” Participants who answered less than 3 times per week were considered to suffer from constipation.

### Data extraction

We used the data on demographic characteristics [age, gender, race, poverty income ratio (PIR), education level]; comorbidities (diabetes and depression); body mass index (BMI); lifestyle characteristics (drinking, smoking, physical activity); dietary intake (total energy, total fat, dietary fiber, moisture); and use of laxatives.

Demographic characteristics (assessed via interview) included age (≤ 40 years, 40–65 years, > 65 years), gender (male, female), race (Non-hispanic White, Non-Hispanic Black, others), PIR (< 1.3, ≥ 1.3, unknown), education level (below high school, high school graduate, college or above).

Comorbidities included diabetes and depression. Diabetes was defined as any of the following: glycated hemoglobin (HbA1c) ≥ 6.5%, fasting blood glucose (FBG) ≥ 126 mg/dL, serum glucose at 2 h following a 75 g glucose load (OGTT) ≥ 200 mg/dL, any self-reported diagnosis of diabetes, or any self-reported use of insulin or other diabetes medication [22]. Depressive symptoms were assessed using Patient Health Questionnaire (PHQ-9), with a total score of 27 points. PHQ-9 score ≥ 10 points or self-reported use of anti-depression agents was defined as depression [23].

BMI was calculated as body weight (kg)/height (m)^2^, which were both measured at the MEC.

Lifestyle characteristics contained drinking, smoking, and physical activity (assessed via interview). Drinking was divided into < 2 times/week and ≥ 2 times/week according to the frequency of drinking. Physical activity was assessed by converting into energy expenditure, which was calculated as recommended metabolic equivalent of task (MET) × exercise time (min). Physical activity was divided into < 450 MET*min/week, ≥ 450 MET*min/week, and unknown [24].

Dietary intake included total energy, total fat, dietary fiber, and moisture. Dietary fiber was the sum of the intake of food and dietary supplements. Moisture (continuous variable) was the sum of the intake of fluid from foods and beverages. The use of laxatives was self-reported.

### Processing of missing data

The missing data were processed through multiple imputation by chained equations (MICE) based on random forest using “miceforest” package in Python 3.9 (Python Software Foundation, Delaware, USA). “Miceforest” package could select which values were imputed by a procedure called predictive mean matching (PMM). PMM involved in the selection of a datapoint from the original, non-missing data which had a predicted value close to the predicted value of the missing sample. The closest N (parameter) values were selected, and one value was chosen from them at random. This could be specified column by column (https://pypi.org/project/miceforest/). According to previously reported study, variables with missing value ≤ 5% (BMI, education level, and smoking) were processed using multiple imputation [25]. Considering that imputation may cause some biases for variables with missing value > 5% [25], the variables with missing value > 5% (PIR, physical activity, and laxative use) were divided into ‘Unknown’ in this study.

### Statistical analysis

To account for complex sampling design of NHANES, data in this study were weighted using appropriate sample weights provided by NHANES. The normality of quantitative data was tested using Kolmogorov-Smirnov. The quantitative data in normalization were represented as mean (standard error) (S.E). Differences between two groups were compared using t test, and differences in more than two groups were compared using analysis of variance. The quantitative data in non-normalization were represented as median and interquartile ranges [M (Q1, Q3)], and differences in more than two groups were compared using Kruskal-Wallis test. Categorical data were shown as number (n) and percentage (%), and differences between groups were compared using chi-squared test. Differences between two groups in rank data were compared using rank sum test.

The association between caffeine intake and constipation was assessed using logistic regression analysis, and results were shown as odds ratio (OR) with 95% confidence intervals (95%CI). For covariates selection, all variables were included into univariate logistic regression model, and the variables with statistical significance were included into multivariable logistic regression. Through stepwise regression, age, gender, PIR, drinking, depression, BMI, dietary fiber, and moisture were finally selected. Crude model represented weighted univariate logistic regression analysis. Adjusted model represented weighted multivariable logistic regression analysis, which adjusted age, gender, PIR, drinking, depression, BMI, dietary fiber, and moisture. Logistic regression analysis was also performed to assess the association between coffee/caffeinated coffee/decaffeinated coffee and constipation.

Sensitivity analysis was performed to avoid bias caused by multiple imputation. Subgroup analysis based on age (age < 40 years: young people; 40 years ≤ age < 65 years: middle-age people; age ≥ 65 years: old people) was performed. Statistical analyses were performed using Python 3.9 and SAS 9.4 (SAS Institute Inc., Cary, NC, USA). *P* < 0.05 was considered to be statistically significant.

## Results

### Selection and characteristics of participants

**Fig. 1:**
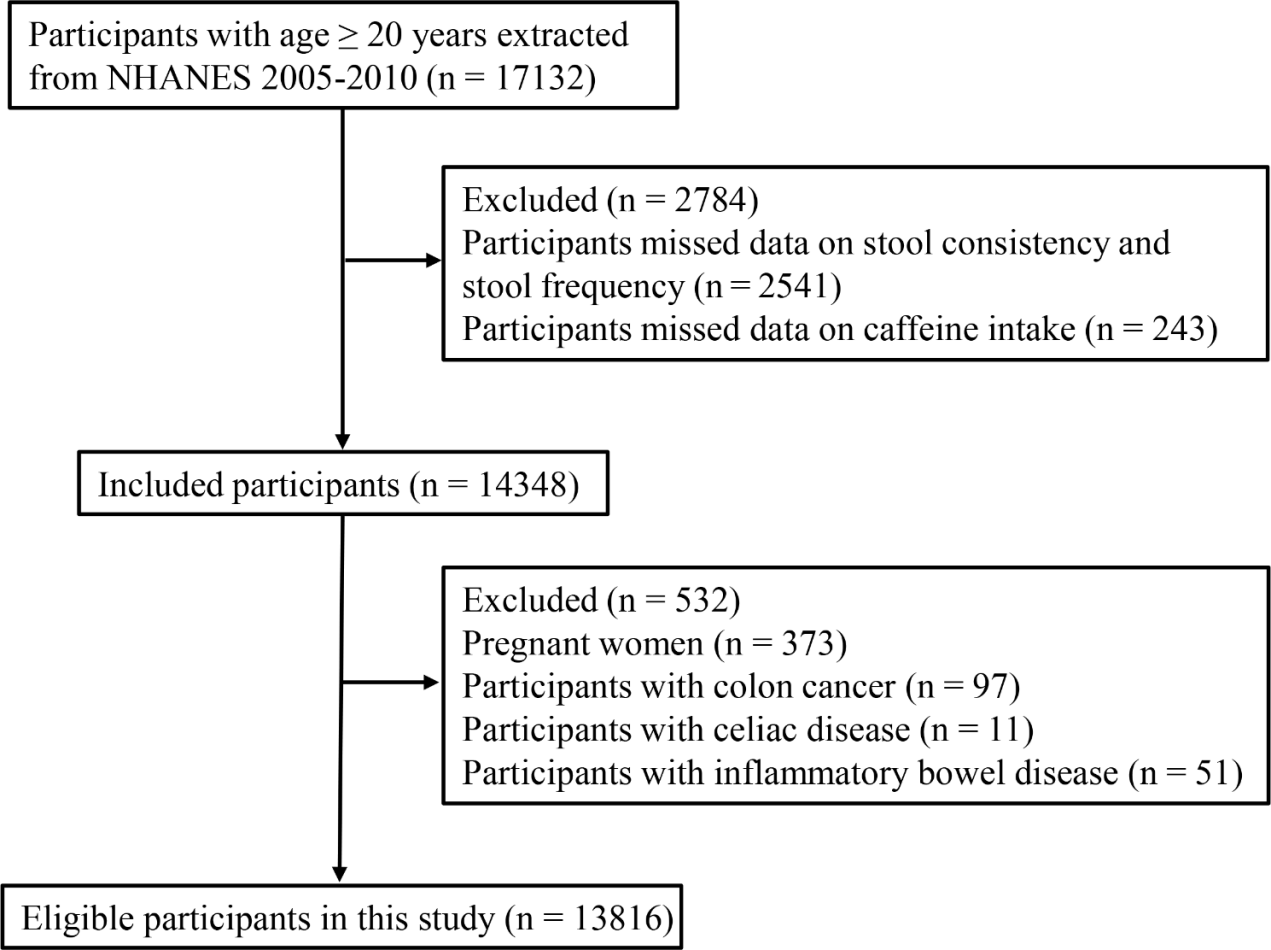
The flowchart of participants selection

A total of 17,132 participants with age ≥ 20 years were extracted from the NHANES database. After excluding participants missing data on stool consistency and stool frequency (n = 2,541) and missing data on caffeine intake (n = 243), 14,348 participants remained. Further, we excluded pregnant women (n = 373), participants with colon cancer (n = 97), with celiac disease (n = 11), and with inflammatory bowel disease (n = 51), 13,816 participants were eligible for analysis (Fig. 1). Supplementary table S1 shows the variables with missing value. Sensitivity analysis showed that estimates did not differ significantly before versus after imputation (Supplementary table S2).

In the eligible participants, 1,413 of them suffered from constipation. The mean caffeine intake was 168 (13.61) mg in the constipation group and 192 (4.57) mg in the non-constipation group (Supplementary table S3). According to quintiles in caffeine intake, the participants were categorized into Q1 (n = 3,204), Q2 (n = 3,098), Q3 (n = 2,859), Q4 (n = 2,453), and Q5 (n = 2,202). Constipation, age, gender, race, PIR, education level, drinking, smoking, diabetes, depression, total energy, total fat, dietary fiber, and moisture were significantly different among the five groups (all *P* < 0.05) (Table 1).

**Table 1:** Characteristics of eligible participants

Variables	Total (n = 13,816)	Caffeine intake					Statistics	*P*
		≤ 10 mg (n = 3204)	10–86 mg (n = 3098)	86–171 mg (n = 2859)	171–303 mg (n = 2453)	> 303 mg (n = 2202)		
Constipation, n (%)							χ^2^ = 42.47	< 0.001
No	12,403 (90.49)	2810 (88.58)	2705 (87.51)	2611 (92.60)	2261 (91.51)	2016 (92.29)		
Yes	1413 (9.51)	394 (11.42)	393 (12.49)	248 (7.40)	192 (8.49)	186 (7.71)		
Age, years. n (%)							χ^2^ = 278.56	< 0.001
≤ 40	4837 (38.65)	1286 (47.79)	1254 (44.93)	981 (38.26)	723 (34.43)	593 (27.78)		
40–65	5843 (45.58)	1146 (33.39)	1156 (39.76)	1197 (45.05)	1134 (49.04)	1210 (60.70)		
> 65	3136 (15.77)	772 (18.82)	688 (15.32)	681 (16.69)	596 (16.54)	399 (11.52)		
Gender, n (%)							χ^2^ = 110.84	< 0.001
Male	6983 (49.03)	1499 (46.02)	1420 (42.33)	1406 (46.47)	1343 (52.12)	1315 (58.23)		
Female	6833 (50.97)	1705 (53.98)	1678 (57.67)	1453 (53.53)	1110 (47.88)	887 (41.77)		
Race, n (%)							χ^2^ = 870.37	< 0.001
Non-Hispanic White	6838 (71.74)	1116 (56.74)	1192 (63.28)	1367 (70.27)	1494 (80.02)	1669 (88.38)		
Non-Hispanic Black	2763 (10.98)	1059 (21.79)	721 (13.77)	509 (10.17)	321 (6.51)	153 (2.66)		
Others	4215 (17.29)	1029 (21.47)	1185 (22.95)	983 (19.56)	638 (13.47)	380 (8.96)		
PIR, n (%)							χ^2^ = 74.70	< 0.001
< 1.3	3746 (18.59)	960 (23.18)	907 (21.23)	744 (18.64)	571 (15.04)	564 (14.86)		
≥ 1.3	9070 (75.80)	2022 (71.95)	1915 (71.33)	1890 (75.15)	1719 (80.05)	1524 (80.55)		
Unknown	1000 (5.61)	222 (4.87)	276 (7.44)	225 (6.21)	163 (4.91)	114 (4.59)		
Education level, n (%)							χ^2^ = 36.17	< 0.001
Below high school	3856 (17.84)	994 (20.24)	989 (20.70)	792 (17.94)	588 (14.39)	493 (15.93)		
High school graduate	3327 (24.55)	715 (21.47)	738 (25.31)	709 (25.59)	591 (25.98)	574 (24.41)		
College or above	6633 (57.61)	1495 (58.29)	1371 (53.99)	1358 (56.48)	1274 (59.63)	1135 (59.66)		
Drinking, n (%)							χ^2^ = 118.59	< 0.001
< 2 times/week	10,792 (73.84)	2603 (79.45)	2553 (79.15)	2266 (75.48)	1765 (66.92)	1605 (68.17)		
≥ 2 times/week	3024 (26.16)	601 (20.55)	545 (20.85)	593 (24.52)	688 (33.08)	597 (31.83)		
Smoking, n (%)							χ^2^ = 235.00	< 0.001
Current non-smokers	10,728 (76.97)	2702 (84.41)	2580 (82.63)	2293 (80.52)	1841 (75.85)	1312 (61.45)		
Current smokers	3088 (23.03)	502 (15.59)	518 (17.37)	566 (19.48)	612 (24.15)	890 (38.55)		
Physical activity, n (%)							χ^2^ = 10.55	0.228
< 450 MET*min/week	1702 (12.75)	388 (11.80)	382 (12.24)	355 (13.91)	304 (12.28)	273 (13.55)		
≥ 450 MET*min/week	7861 (61.94)	1814 (61.85)	1705 (61.19)	1607 (60.88)	1466 (64.83)	1269 (60.97)		
Unknown	4253 (25.30)	1002 (26.36)	1011 (26.57)	897 (25.21)	683 (22.89)	660 (25.48)		
Diabetes, n (%)							χ^2^ = 35.24	< 0.001
No	9680 (75.53)	2230 (75.42)	2194 (76.40)	1961 (73.44)	1700 (73.94)	1595 (78.44)		
Treated	3499 (21.14)	820 (20.76)	749 (19.88)	743 (22.17)	648 (22.91)	539 (20.02)		
Untreated	637 (3.32)	154 (3.83)	155 (3.72)	155 (4.40)	105 (3.15)	68 (1.54)		
Depression, n (%)							χ^2^ = 14.29	0.006
No	11,528 (82.72)	2737 (85.04)	2626 (83.45)	2410 (83.43)	2023 (81.36)	1732 (80.34)		
Yes	2288 (17.28)	467 (14.96)	472 (16.55)	449 (16.57)	430 (18.64)	470 (19.66)		
BMI, kg/m^2^, Mean (S.E)	28.71 (0.12)	28.92 (0.25)	28.56 (0.14)	28.59 (0.20)	28.97 (0.19)	28.52 (0.16)	F = 1.87	0.132
Total energy, kcal, Mean (S.E)	2165.16 (16.15)	1989.67 (27.34)	2063.55 (25.80)	2115.03 (29.37)	2204.52 (25.10)	2452.81 (39.44)	F = 26.25	< 0.001
Total fat, gm, Mean (S.E)	82.12 (0.83)	72.89 (1.39)	77.24 (1.33)	79.73 (1.42)	85.21 (1.25)	95.50 (1.77)	F = 30.00	< 0.001
Dietary fiber, gm, Mean (S.E)	16.43 (0.23)	16.16 (0.31)	15.72 (0.27)	16.57 (0.40)	16.47 (0.34)	17.24 (0.37)	F = 4.34	0.005
Moisture, gm, Mean (S.E)	3088.80 (29.72)	2764.83 (42.62)	2597.81 (41.33)	2872.51 (36.57)	3195.06 (39.89)	4014.84 (50.09)	F = 149.61	< 0.001
Laxative use, n (%)							χ^2^ = 10.11	0.258
No	4464 (30.04)	1006 (29.27)	994 (31.50)	959 (30.77)	811 (30.62)	694 (28.05)		
Yes	555 (3.26)	153 (4.12)	120 (3.25)	121 (3.37)	90 (2.74)	71 (2.84)		
Unknown	8797 (66.69)	2045 (66.61)	1984 (65.25)	1779 (65.86)	1552 (66.63)	1437 (69.11)		

Abbreviation: Mean (S.E), mean (standard error); PIR, poverty income ratio; MET, metabolic equivalent of task; BMI, body mass index

### Association between caffeine intake and constipation

Logistic regression analysis was used to assess the association between caffeine intake and constipation. In the crude model, caffeine intake in the Q3, Q4, and Q5 was associated with the lower odds of constipation than the caffeine intake in the Q1, with OR of 0.62 (95%CI: 0.50–0.76), 0.72 (95%CI: 0.55–0.93), and 0.65 (95%CI: 0.51–0.83), respectively. After adjusting age, gender, PIR, drinking, depression, BMI, dietary fiber, and moisture, we also found that higher intake of caffeine was associated with the lower odds of constipation (Q3: OR = 0.60, 95%CI: 0.49–0.74; Q4: OR = 0.77, 95%CI: 0.59–0.99; Q5: OR = 0.72, 95%CI: 0.56–0.92) (Table 2).

**Table 2:** Association between caffeine intake and constipation

Caffeine intake	Number	Crude model		Adjusted model	
		**OR** (**95% CI**)	***P***	**OR (95% CI)**	***P***
Q1	3204	Ref		Ref	
Q2	3098	1.11 (0.93–1.32)	0.252	0.99 (0.82–1.21)	0.954
Q3	2859	0.62 (0.50–0.76)	< 0.001	0.60 (0.49–0.74)	< 0.001
Q4	2453	0.72 (0.55–0.93)	0.011	0.77 (0.59–0.99)	0.047
Q5	2202	0.65 (0.51–0.83)	< 0.001	0.72 (0.56–0.92)	0.009

Abbreviation: OR, odds ratio; CI, confidence interval

Crude model: weighted univariate logistic regression analysis

Adjusted model: weighted multivariable logistic regression analysis adjusted age, gender, PIR, drinking, depression, BMI, dietary fiber, and moisture

Supplementary table S4 shows that higher intake of coffee was associated with the lower odds of constipation (502–754 g vs. 0 g: OR = 0.67, 95%CI: 0.52–0.85; > 754 g vs. 0 g: OR = 0.67, 95%CI: 0.51–0.89) in the adjusted model. Further, we explored the association between caffeinated coffee or decaffeinated coffee and constipation. Results showed that patients drinking caffeinated coffee had lower odds of constipation than those who with no intake of caffeinated coffee after adjusting age, gender, PIR, drinking, depression, BMI, dietary fiber, and moisture (*P* < 0.05), while there was no statistically significant association between decaffeinated coffee and constipation (Supplementary table S5).

### Association between caffeine intake and constipation based on age

The association between caffeine intake and constipation based on age was explored. After adjusting gender, PIR, drinking, depression, BMI, dietary fiber, and moisture, statistically significant association was found between caffeine intake and constipation in young participants (Q3: OR = 0.47, 95%CI: 0.31–0.70). For middle-age participants, caffeine intake in the Q3 (OR = 0.61, 95%CI: 0.43–0.86), Q4 (OR = 0.61, 95%CI: 0.38–0.98), and Q5 (OR = 0.67, 95%CI: 0.45–0.99) was significantly associated with the lower odds of constipation. There was no statistically significant association between caffeine intake and constipation in old participants (*P* > 0.05) (Table 3).

**Table 3:** Association between caffeine intake and constipation based on age

Variables	Number	Crude model		Adjusted model	
		**OR** (95% ***CI***)	***P***	**OR (95% CI)**	***P***
Age < 40 years					
Q1	1286	Ref		Ref	
Q2	1254	1.21 (0.86–1.69)	0.265	1.05 (0.75–1.48)	0.768
Q3	981	0.50 (0.35–0.73)	< 0.001	0.47 (0.31–0.70)	< 0.001
Q4	723	0.89 (0.65–1.23)	0.470	0.86 (0.61–1.19)	0.352
Q5	593	0.67 (0.42–1.08)	0.098	0.68 (0.42–1.10)	0.116
40 years ≤ age < 65 years					
Q1	1146	Ref		Ref	
Q2	1156	1.02 (0.72–1.44)	0.924	0.89 (0.63–1.26)	0.499
Q3	1197	0.67 (0.47–0.95)	0.026	0.61 (0.43–0.86)	0.006
Q4	1134	0.60 (0.36–0.99)	0.047	0.61 (0.38–0.98)	0.042
Q5	1210	0.65 (0.44–0.96)	0.031	0.67 (0.45–0.99)	0.048
Age ≥ 65 years					
Q1	772	Ref		Ref	
Q2	688	1.03 (0.69–1.52)	0.889	0.97 (0.63–1.47)	0.868
Q3	681	0.83 (0.58–1.18)	0.291	0.81 (0.56–1.18)	0.263
Q4	596	0.76 (0.47–1.22)	0.248	0.84 (0.52–1.37)	0.486
Q5	399	0.61 (0.35–1.07)	0.084	0.69 (0.39–1.24)	0.207

Abbreviation: OR, odds ratio; CI, confidence interval

Crude model: weighted univariate logistic regression analysis

Adjusted model: weighted multivariable logistic regression analysis adjusted gender, PIR, drinking, depression, BMI, dietary fiber, and moisture

## Discussion

In this study, we explored the association between caffeine intake and constipation in participants from the NHANES database with a large nationally representative sample. We found that high caffeine intake was associated with the low odds of constipation after adjusting a wide range of potential confounders. The similar association was found in young and middle-age participants. Further, we found that drinking caffeinated coffee was associated with the low odds of constipation. There was no statistically significant association between decaffeinated coffee and constipation.

The high prevalence of constipation brings big burden to medical system and impairs people’s quality of life [4–6]. Colonic motor dysfunction is one of the reasons for constipation [6]. It was well known that caffeine played an important role in the motor activity of gastrointestinal tract [12, 26–28]. Rao et al. have found that caffeinated coffee induced greater motor activity in the transverse/descending colon, and decaffeinated coffee did not affect the colonic motility [29]. Brown et al. have reported that caffeinated coffee stimulated a motor response of the distal colon in some people, and tea (caffeine content 1.5-3%) had a similar effect to coffee [26]. A study in Thailand showed that caffeine intake caused a decrease of rectal sensory threshold in the defecation desire, leading to an earlier desire to defecate [27]. Colonic microbiota disturbance was another possible reason for constipation [6, 30]. González et al. have found that long-term consumption of coffee seemed to be associated with changes of certain intestinal microbiota in which caffeine may play a role [13]. A cross-sectional study indicated that coffee consumption induced changes in the intestinal microbial composition of Saudis [14]. In this study, we found that high intake of caffeine was associated with the low odds of constipation. There are several explanations for this. First, caffeine may increase gastrointestinal motility, stimulate the movement response of distal colon, and induce an earlier desire of defecation, thereby lowering the odds of constipation [31]. Second, caffeine may increase the activity of probiotics, which is beneficial for gut motility [32, 33]. We also found that caffeinated coffee was associated with the constipation, while no significant association was found between decaffeinated coffee and constipation, which further indicated the association between caffeine and constipation. In the future, prospective studies should be performed to clarify the association between caffeine intake and constipation.

Evidence has shown that increasing age was associated with the increased risk of constipation [6]. Constipation was common in the older adults; inadequate fiber or fluid intake, and less physical activity may induce the constipation in the old people, with the prevalence ranging from 9 to 60% [34, 35]. In our study, the association between caffeine intake and constipation was not found in the old people. This may be because that the number of old people in our study was relatively small (15.77%), which may not have enough statistical power to support the significant results. Future studies with larger sample size of old people are needed to verify this finding. In the young and middle-age people, we found that high caffeine intake was associated with lower odds of constipation. Our findings indicated that caffeine intake may be important for the management of constipation in the young and middle-age people.

### Implications

We found the association between high caffeine intake and the low odds of constipation. Caffeine exhibits the potential in regulating colonic motor dysfunction and colonic microbiota disturbance, which are the potential causes of constipation [13, 28, 32]. Management measures that influence caffeine intake may be a target for public health to prevent and relief the constipation. For individuals without constipation, we suggest they develop a consciousness of moderately consuming caffeinated foods to prevent constipation. For patients with constipation, we suggest they develop a habit of consuming caffeinated foods and drinks, such as coffee, tea, and chocolate bar, to mitigate constipation.

### Strengths and limitations

Our study includes a relatively larger sample size to explore the association between caffeine intake and constipation. The sample size is extracted from the NHANES, which is a large and nationally representative database that supports the generalizability of our findings in the U.S. adult population. To avoid bias caused by multiple imputation, sensitivity analysis is performed, which expands the robustness of results. In addition, subgroup analysis is performed based on age to further determine the association between caffeine intake and constipation in different populations. Some limitations exist in this study. First, this is a cross-sectional study, which is unable to infer causality. The association between caffeine intake and constipation should be further explored by prospective studies and randomized, controlled trials in the future. Second, despite potential confounders affecting constipation we have adjusted, some confounders may still not be considered due to the limitation of the database. Third, data on dietary intake are obtained through the 24-h dietary recall, which may be subject to measurement error and might not reflect a person’s usual diet. Fourth, physical activity and laxative use are important covariates, but both of them are substantial missing and grouped as “unknown”, which may not adequately address possible confounding. Our findings should be cautiously interpreted.

## Conclusion

This study found that high caffeine intake was associated with low odds of constipation. The similar association was found in the young and middle-age people. Our findings indicated that caffeine intake in daily may be helpful in the management of constipation. Individuals without constipation should develop a consciousness to moderately consume caffeinated foods for the prevention of constipation. Patients with constipation should develop a habit to consume caffeinated foods and drinks for the mitigation of constipation. In the future, prospective studies are needed to elucidate the association between caffeine intake and constipation.

### Electronic supplementary material

Below is the link to the electronic supplementary material.

Supplementary Material 1

Supplementary Material 2

## Data Availability

The datasets generated and/or analyzed during the current study are available in the NHANES database, https://wwwn.cdc.gov/nchs/nhanes/.

## Abbreviations

NHANES: National Health and Nutrition Examination Survey
NCHS: National Center for Health Statistics
MEC: Mobile Examination Center
USDA: U.S Department of Agriculture
FNDDS: Food and Nutrient Database for Dietary Studies
PIR: Poverty income ratio
BMI: Body mass index
HbA1c: Glycated hemoglobin
FBG: Fasting blood glucose
PHQ-9: Patient Health Questionnaire
