# Supplementary material for: Exploring the connection between caffeine intake and constipation: a cross-sectional study using national health and nutrition examination survey data
Source: BMC Public Health. 2024 Jan 2;24:3. doi: 10.1186/s12889-023-17502-w (PMC10759350; doi:10.1186/s12889-023-17502-w)
Supplement: Supplementary file 1 — Supplementary Material 1 [file 12889_2023_17502_MOESM1_ESM.docx]

**Supplementary table S1 Variables and percentage of missing data**

| **Variables** | **N (%)** |
| --- | --- |
| PIR | 1000 (7.24%) |
| BMI | 120 (0.87%) |
| Education level | 12 (0.09%) |
| Smoking | 3 (0.02%) |
| Physical activity | 4253 (30.78%) |
| Laxative use | 8797 (63.67%) |

Abbreviation: N, number; PIR, poverty income ratio; BMI, body mass index.

**Supplementary table S2 Sensitivity analysis for multiple imputation**

| **Variables** | **Before imputation** | **After imputation** | **Statistics** | ***P*** |
| --- | --- | --- | --- | --- |
| BMI, kg/m^2^, Mean (S.E) | 28.69 (0.12) | 28.71 (0.12) | t = -1.92 | 0.061 |
| Education level, n (%) |  |  | χ^2^ = 5.59 | 0.232 |
| Less than 9th grade | 1633 (5.74) | 1636 (5.74) |  |  |
| 9-11th grade | 2220 (12.11) | 2220 (12.10) |  |  |
| High school graduate/GED or equivalent | 3323 (24.53) | 3327 (24.55) |  |  |
| Some college or AA degree | 3845 (30.95) | 3850 (30.96) |  |  |
| College graduate or above | 2783 (26.66) | 2783 (26.64) |  |  |
| Smoking, n (%) |  |  | χ^2^ = 2.36 | 0.124 |
| No | 10725 (76.96) | 10728 (76.97) |  |  |
| Yes | 3088 (23.04) | 3088 (23.03) |  |  |

Abbreviation: BMI, body mass index; Mean (S.E), mean (standard error); GED, General Educational Development; AA, Associate of Arts.

**Supplementary table S3 Caffeine intake in constipation group and non-constipation group**

| **Variables** | **Total (n=13816)** | **Constipation (n=1413)** | **Non-constipation (n=12403)** | **Statistics** | ***P*** |
| --- | --- | --- | --- | --- | --- |
| Caffeine^1^, mg, Mean (S.E) | 190 (4.65) | 168 (13.65) | 192 (4.58) | t = 1.83 | 0.073 |
| Caffeine^2^, mg, Mean (S.E) | 190 (4.64) | 168 (13.61) | 192 (4.57) | t = 1.84 | 0.072 |

Abbreviation: Mean (S.E), mean (standard error).

Note: ^1^ Caffeine intake was from food and beverages; ^2^ Caffeine intake was from food, beverages, and dietary supplements.

**Supplementary table S4 Association between coffee and constipation**

| **Coffee intake** | **Number (percentage)** | **OR (95% CI)** | ***P*** |
| --- | --- | --- | --- |
| 0 g | 6470 (46.73%) | Ref |  |
| 0-311 g | 2286 (13.49%) | 0.85 (0.71-1.03) | 0.091 |
| 311-502 g | 1683 (11.50%) | 0.80 (0.58-1.12) | 0.195 |
| 502-754 g | 1736 (13.61%) | 0.67 (0.52-0.85) | 0.002 |
| > 754 g | 1641 (14.67%) | 0.67 (0.51-0.89) | 0.006 |

Abbreviation: OR, odds ratio; CI, confidence interval.

Note: weighted multivariable logistic regression analysis adjusted age, gender, PIR, drinking, depression, BMI, dietary fiber, and moisture.

**Supplementary table S5 Association between caffeinated or decaffeinated coffee and constipation**

| **Coffee intake** | **Number (percentage)** | **OR (95% CI)** | ***P*** |
| --- | --- | --- | --- |
| Caffeinated coffee |  |  |  |
| 0 g | 7663 (53.86%) | Ref |  |
| 0-310 g | 1818 (11.25%) | 0.79 (0.65-0.95) | 0.014 |
| 310-502 g | 1460 (10.33%) | 0.72 (0.52-0.99) | 0.046 |
| 502-754 g | 1466 (11.92%) | 0.71 (0.55-0.91) | 0.008 |
| > 754 g | 1409 (12.64%) | 0.72 (0.54-0.96) | 0.024 |
| Decaffeinated coffee |  |  |  |
| 0 g | 12444 (91.44%) | Ref |  |
| 0-250 g | 302 (1.55%) | 1.31 (0.80-2.14) | 0.279 |
| 250-357 g | 426 (2.53%) | 1.26 (0.77-2.06) | 0.355 |
| 357-700 g | 384 (2.31%) | 0.98 (0.61-1.55) | 0.914 |
| > 754 g | 260 (2.17%) | 0.48 (0.22-1.01) | 0.054 |

Abbreviation: OR, odds ratio; CI, confidence interval.

Note: weighted multivariable logistic regression analysis adjusted age, gender, PIR, drinking, depression, BMI, dietary fiber, and moisture.
