# Supplementary figures and images for: Exploring the connection between caffeine intake and constipation: a cross-sectional study using national health and nutrition examination survey data
Source: BMC Public Health. 2024 Jan 2;24:3. doi: 10.1186/s12889-023-17502-w (PMC10759350; doi:10.1186/s12889-023-17502-w)

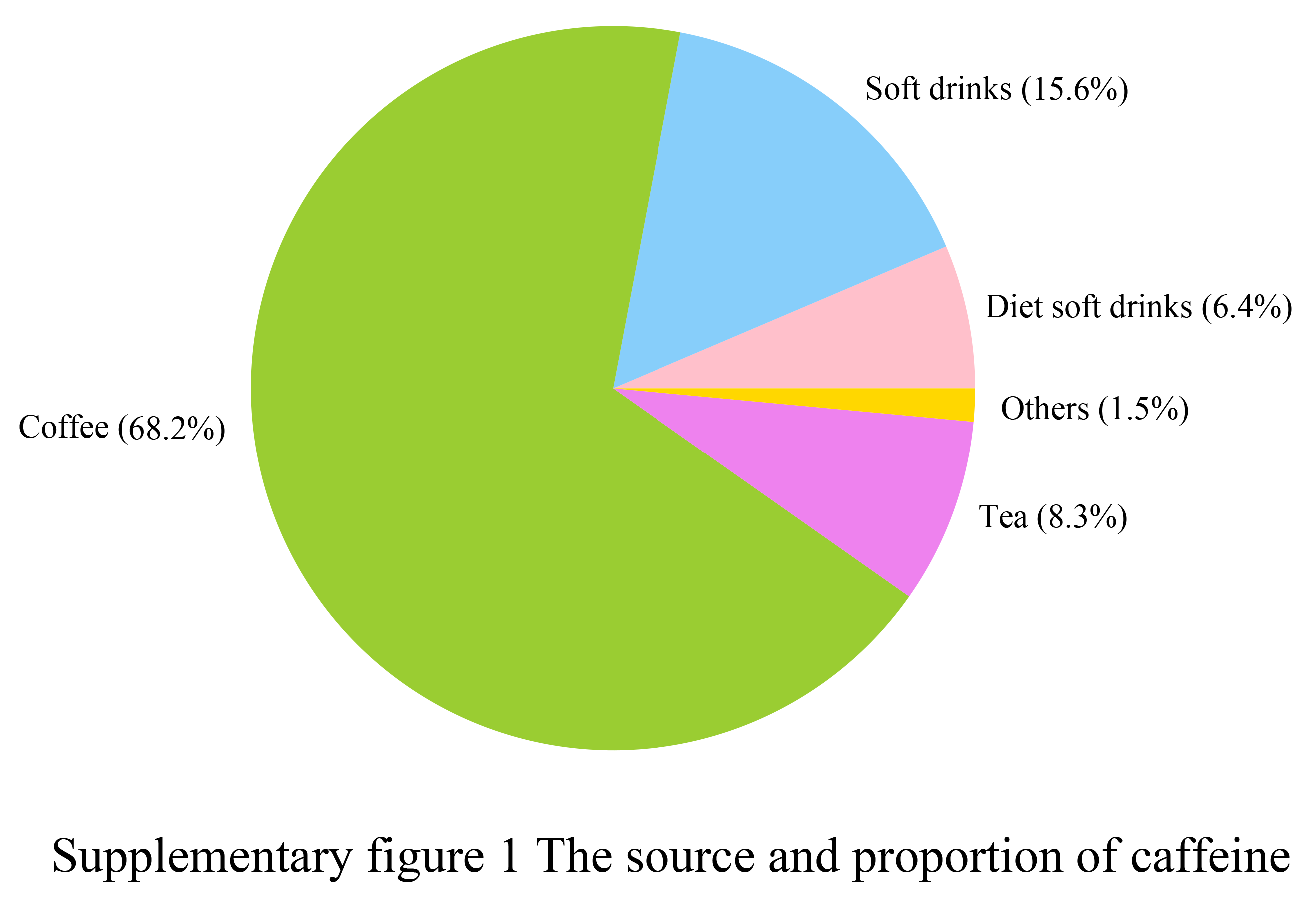

Supplement: Supplementary file 2 — Supplementary Material 2 [file 12889_2023_17502_MOESM2_ESM.tif]
